# Readability of online COVID-19 health information: a comparison between four English speaking countries

**DOI:** 10.1186/s12889-020-09710-5

**Published:** 2020-11-13

**Authors:** Amy P. Worrall, Mary J. Connolly, Aine O’Neill, Murray O’Doherty, Kenneth P. Thornton, Cora McNally, Samuel J. McConkey, Eoghan de Barra

**Affiliations:** 1 Department of Infectious Diseases, Beaumont Hospital, Dublin, 9 Ireland; 2 Royal College of Surgeons Ireland, Dublin, Ireland; 3 Department of Surgery, Beaumont Hospital, Dublin, Ireland; 4 Department of International Health and Tropical Medicine, Royal College of Surgeons Ireland, Dublin, Ireland

**Keywords:** COVID-19, Coronarvirus pandemic, Health information, Readability, Health literacy

## Abstract

**Background:**

The internet is now the first line source of health information for many people worldwide. In the current Coronavirus Disease 2019 (COVID-19) global pandemic, health information is being produced, revised, updated and disseminated at an increasingly rapid rate. The general public are faced with a plethora of misinformation regarding COVID-19 and the readability of online information has an impact on their understanding of the disease. The accessibility of online healthcare information relating to COVID-19 is unknown. We sought to evaluate the readability of online information relating to COVID-19 in four English speaking regions: Ireland, the United Kingdom, Canada and the United States, and compare readability of website source provenance and regional origin.

**Methods:**

The Google® search engine was used to collate the first 20 webpage URLs for three individual searches for ‘COVID’, ‘COVID-19’, and ‘coronavirus’ from Ireland, the United Kingdom, Canada and the United States. The Gunning Fog Index (GFI), Flesch-Kincaid Grade (FKG) Score, Flesch Reading Ease Score (FRES), Simple Measure of Gobbledygook (SMOG) score were calculated to assess the readability.

**Results:**

There were poor levels of readability webpages reviewed, with only 17.2% of webpages at a universally readable level. There was a significant difference in readability between the different webpages based on their information source (*p* < 0.01). Public Health organisations and Government organisations provided the most readable COVID-19 material, while digital media sources were significantly less readable. There were no significant differences in readability between regions.

**Conclusion:**

Much of the general public have relied on online information during the pandemic. Information on COVID-19 should be made more readable, and those writing webpages and information tools should ensure universal accessibility is considered in their production. Governments and healthcare practitioners should have an awareness of the online sources of information available, and ensure that readability of our own productions is at a universally readable level which will increase understanding and adherence to health guidelines.

## Background

The Coronavirus Disease 2019 (COVID-19) pandemic has led to an expected increase in the number of online searches on the condition. Internet users are now frequently searching for health related information and as a tool to answer questions about symptoms, diagnoses and treatment [1]. Social distancing, lockdowns and self-isolation policies worldwide have also meant patients’ access to in-person health care advice has decreased and reliance on either telemedicine or online information has increased. This is reflected in the rise of Google® Trends searches for ‘coronavirus’, ‘COVID’ and ‘COVID-19′ in recent months [2].

The internet as a source of health information is unregulated and the quality, reliability, and accessibility to the reader is variable. While there are some quality guidelines available, such as Health on the Net (www.hon.ch/en), which promotes reliable and transparent health information online, there is little guidance for readability of online health information [3]. Many webpages provide inaccurate or questionable information and this can be harmful [4]. A small number of studies have already reported on the quality of COVID-19 related health information [5], and indeed the misinformation that has appeared on webpages and in particular on social media in recent months [4, 6]. The quality of information relating to COVID-19 accessed found that there are often discrepancies between health information issued by public health organisation and general information available on other digital media [7].

Several tools are available to assess the readability of information, such as the Gunning Fox Index (GFI), the Flesch Reading Ease Score (FRES), the Flesch-Kincaid Grade (FKG) and the Simple Measure of Gobbledygook (SMOG) score [8]. These tools are established validated readability tools and are validated in health information studies and the English language, and have defined score levels for universal readability [9]. The readability of health information related to COVID-19 has not been published. We sought to evaluate the readability of online information relating to COVID-19 in four English speaking regions: Ireland, the United Kingdom, Canada and the United States, ranking of websites, and compare readability of website source provenance and regional origin.

## Methods

### Webpage search and identification

The Google® search engine was used to collate the first 20 webpage URLs for three individual searches for ‘COVID’, ‘COVID-19’, and ‘coronavirus’. When searching for information on the internet users typically will pick one of the first five search results, and will typically rephrase their search criteria instead of proceeding to the second page (or further) [10], as a result we only included results from the first page of search engine results. The searches were conducted from geolocation search engine settings, in web-browser Google Chrome Version 85, to reflect the webpages found in Ireland, the United Kingdom, Canada and the United States. All searches were conducted on 17th April 2020. All previous search history and data caches were cleared before the first search, and between searches. Webpage results are tabulated in Appendix 1. Results were categorised by two researchers (AW and MC) independently based on source provenance of the webpage; ‘government and public health organisations’, ‘educational or scientific institution’, ‘digital media’ or ‘other’. A fifth category of ‘peer-reviewed journals/articles’ was included, but no webpage results fell into this category, and as such we have not included it in results. Source provenance for ‘government and public health organisations’ required that the webpage was supported, funded or hosted on a government, state, county or federal website platform (.gov.us, .gov.nl.ca, hse.ie, nhs.co.uk, as some examples), ‘educational or scientific institutions’ included sources such as Mayo Clinic, Medline, WebMD, etc., ‘digital media’ sources were webpages from news outlets, newspaper digital platforms etc., and ‘other’ captured the remaining webpages that fell out of these categories, similar to previous published categories in readability analyses [11].

### Readability assessment tools

Four scores were used to calculate readability of the webpages; the Gunning Fog Index (GFI), the Flesch Kincaid Grade (FKG) Score and Flesch Reading Ease Score (FRES) and the Simple Measure of Gobbledygook (SMOG) Index. To ensure consistency and avoid human error the readability tests were done using an online readability calculator to provide FRES, FKG, GFI and SMOG scores [12]. All webpages were screened by the readability tool and hyperlinks, non-standard text, abbreviations and author names were not included in the analysis to prevent low-skewing of results.

### The Flesch Reading Ease Score (FRES)

The FRES is a tool that indicates readability of English text on a 100-point scale. The FRES can be calculated using the following formula: [206.835 – (1.015 x (total words ÷ total sentences)) – (84.6 x (total syllables ÷ total words))]. The higher the score the greater the ease of comprehension, e.g. > 90 scores indicate something that would easily be understood by a 10–11 year old. A recommended score between 60 and 70 represents a suitable readability level for most 13 year olds, which adequately captures most patient cohorts [13].

### The Flesch-Kincaid Grade Score

The Flesch-Kincaid Grade (FKG) Score is a readability test used extensively in educational settings, it gives a marker of readability with a weighting on syllables. It can be calculated with the following formula: 0.39 (total words/total sentences) + 11.8 (total syllables/total words) – 15.59. The resulting number gives an estimated United States grade level equivalent. For universal accessibility and readability a suitable score is < 8.

### The Gunning Fox Index

The GFI tool is an English language tool measuring readability by estimating years of formal education needed to understand a text on the first time of reading. The GFI can be calculated using the following formula: 0.4 x [(words ÷ sentences) + 100 x (complex words ÷ total words)]. A lower score indicates sample text that is more easily read. The GFI scale runs from 6 to 17; where 6 represents the reading level of an 11–12 year old, 12 is an 18 year old who has completed second level education, and 17 is a university level graduate [13]. Information requiring near universal readability should have a GFI < 8 [14].

### The Simple Measure of Gobbledygook Index

The SMOG readability index estimates the number of years of formal education that a reader would need in order to read the material tested. The SMOG formula is: 3 + square root √ [number of polysyllabic words x (30 ÷ number of sentences)]. SMOG is only validated in the English language and is validated in healthcare information studies [15]. A suitable SMOG score for universal readability is 10.

### Statistical analysis

Descriptive statistics were calculated for SMOG, FRES, FKG and GFI scores. Shapiro-Wilk test determined parametric or non-parametric data distribution. Mean (SD) were used for normally distributed data, while median (range) were used for non-parametrically distributed data. Spearman’s correlations and Pearson’s correlations were used to assess non-parametric and parametric association between readability scores respectively. ANOVAs and Kruskall-Wallis tests were used to compare differences between the mean or median readability scores for univariate group analysis to determine differences between country, continent and source provenance. A 5% level of significance was used for all statistical tests. All statistical analysis was performed using GraphPad Prism software Version 8 (La Jolla, CA, USA, 2020), SPSS Statistics Version 26 (IBM, 2020) and Microsoft Office Excel Version 16 (USA, 2018).

## Results

The searches were performed using the keywords: coronavirus, COVID, COVID-19. The first 20 webpages were collated from each search and the search was conducted geolocated to Ireland, the United Kingdom, Canada and the United States, totalling 240 webpages (Appendix 1). Of the 240 webpages analysed 53% (*n* = 127) were government organisations or public health organisation webpages, 29% (*n* = 69) were digital or social media webpages, 5% (*n* = 11) were from scientific or educational institutions and 14% (*n* = 33) were from other sources (Table 1A). There was a relation by chi squared analysis between country and source type of information in the website results (x^2^ = 23.69, *p* < 0.00481). This relationship was investigated for differences between regional spread of webpage sources (ANOVA, *p* < 0.042), with Canada and the United States having higher numbers of public health and governmental websites than Ireland and the United Kingdom (Table 1A). There was matching inverse correlation between webpage sources between countries (r − 0.172, 95% CI [− 0.2960 to − 0.04293], *p* < 0.007,) and between continents (r − 0.185, 95% CI [− 0.3084 to − 0.05652], *p* < 0.0039), both by non-parametric Spearman correlation analysis.

**Table 1:** A) Webpages tabulated by country and source type. B) Readability scores for webpages by region. Statistical analysis showed no significant difference between countries or continents. FRES target score > 60, FKG target < 8, GFI target < 8 and SMOG target score < 10. C) Readability scores for webpages by source type. ANOVA for normally distributed FRES with mean (SD), and Kruskal-Wallis for non-parametric FKG, GFI and SMOG scores with median (range)

**A**	**Government and Public Health Organisations**	**Educational or Scientific Institution**	**Digital Media**	**Other**
**Ireland**	29	2	16	13
**United Kingdom**	22	7	24	7
**Canada**	39	1	13	9
**United States**	37	1	16	4
**Total**	**127**	**11**	**69**	**33**
**B**	**FRES** **Mean (SD)**	**FKG** **Median (range)**	**GFI** **Median (range)**	**SMOG** **Median (range)**
**Ireland**	48.93 (12.45)	8.7 (5–14)	8.7 (3–16)	10.4 (7–17)
**United Kingdom**	52.21 (13.04)	8.7 (4–20)	9.25 (4–18)	10.9 (9–18)
**Canada**	47.89 (9.57)	8.8 (4–14)	8.8 (2–15)	11.0 (7–15)
**United States**	49.58 (10.25)	8.8 (5–20)	8.6 (3–18)	11.0 (7–18)
**Total**	**49.65 (11.4)**	**8.8 (4–20)**	**8.8 (2–18)**	**10.9 (7–18)**
**C**	**FRES** **Mean (SD)**	**FKG** **Median (range)**	**GFI** **Median (range)**	**SMOG** **Median (range)**
**Government and Public Health Organisations**	48.5 (13.1)	8.7 (4–20)	8.7 (3–18)	10.4 (7–18)
**Educational or Scientific Institution**	44.8 (10.1)	10.4 (8–14)	11.1 (8–15)	11.7 (10–15)
**Digital Media**	53.3 (10.6)	9.4 (4–14)	9.7 (2–15)	11.2 (8–14)
**Other**	47.52 (10.89)	8.6 (5–12)	8.0 (3–14)	11.1 (9–14)
**Univariate Analysis (*p*-value)**	< 0.0196*	< 0.04*	< 0.0003***	< 0.0009***

* *p* ≤ 0.05, ** *p* ≤ 0.01, *** *p* ≤ 0.001, ****p ≤ 0.0001

FRES results were parametric, while FKG, GFI and SMOG scores were all non-parametric. Only 17.2% (*n* = 165) of all the readability scores analysed demonstrated a universally readable level. 19% (*n* = 45) of FRES scores were at a universally readable level (> 60), 32% (*n* = 77) of FKG scores (target < 8), 37% (*n* = 88) of GFI scores (target < 8), and only 30% (*n* = 73) of SMOG scores were at a universally readable levels (< 10). The mean readability scores for webpages searched from all regions were below the standard universal readability levels, and there were no significant differences comparably between regions (Table 1B).

There were significant differences between the readability of webpages depending on the information source for all readability scores FRES (*p* < 0.0196), FKG (*p* < 0.04), GFI (*p* < 0.0003), and SMOG (*p* < 0.0009) by ANOVA analyses (Table 1C). From this analysis the most readable sources across the majority of the scores were webpages issued by government and public health organisations. All four readability scores (FRES, FKG, GFI and SMOG) correlated with each other significantly (Spearman’s correlations, r values, *p* values and 95% CI available in Appendix 2). There was a positive association between source of information category and ranking of the webpage on the search engine results by Spearman correlation (r 0.184, 95% CI [0.05525 to 0.3072], *p* < 0.004).

## Discussion

Health literacy is an important barrier for communication by health professionals, public health bodies and government institutions with the public [9]. The COVID-19 pandemic presents a number of health literacy obstacles which include the rapid publication of information, the frequently evolving and fluctuating nature of public guidelines and health information, the lack of specific treatments, with an evidence base, for COVID-19 pneumonia, and the inconsistent and sometimes dangerous information and misinformation that is occurring online, in particular on social media [16]. Basic access to reliable, high quality and readable online information is an economic and social privilege, and the COVID-19 pandemic has highlighted this digital inequality [17]. Indeed, readability of online health information related to other epidemics such as Zika virus, and Ebola virus disease also found the majority of health information, including governmental and public health sources to be beyond basic readability levels [18, 19]. A fundamental necessity to understanding and engaging with health information is the accessibility and readability of the information and while there is a pressure and immediacy to publish information at short notice, readability should be considered when producing health literature and information [16].

The webpages analysed were mostly higher than an acceptable universal level for readability. The universal level of readability is generally accepted to be that of a child, aged 10–11 that has attended primary school or junior school [13]. The best performing readability score found only 37% of webpages readable to a universal audience, this does not reflect well for the health information produced and disseminated online. Similar studies of quality and readability of online health information also often report poor readability levels including in vascular surgery [20], respiratory medicine [11], and genitourinary medicine [21]. This poor readability level affects understanding of the health information; resulting in poor adherence to hygiene measures, social-distancing measures, and further public health recommendations [6].

Webpages most likely to be viewed are webpages on the first page of search results [10], making website rankings an important factor for consideration [22]. Our analysis included only webpages from the first page of search engine results and the moderately positive correlation between source type and ranking of webpages on the search results is reassuring as the majority of webpages were published by public health organisations or government bodies, and they tended to be both ranked higher on the results list and have better readability scores.

Search engines have the ability to manipulate ranking settings, and sponsored search results can often tamper with what audiences see first [22]. Google®, has been making corporate decisions to artificially rank high-profile health information from respectable prevalence such as the World Health Organisation since early March 2020 [23]. This might explain why Government and Public Health bodies account for 53% (*n* = 127) of search results, and while this is reassuring because readability tends to be higher from information from those origins, the mean readability scores in this study remain poor. These differences seen between countries and continents in both the type of source information available is worth considering, given that there is a clear difference in readability between sources.

The correlations between the various readability scores was reassuring and showed that while there are some differences that the trend in detecting poor readability was similar between tests (Appendix 2). While much has been published in the last few weeks on the quality of health information and the misinformation relating to COVID-19, this is the first assessment of readability of online information on COVID-19 with comparisons between four English speaking countries.

We acknowledge the limitations of this study. There are a number of weaknesses associated with each of the readability scores [14, 24]. The tests rely on numbers of words in sentences, or syllables in words, which may not always reflect the reading level. The scores do not consider layout, infographics or figures that often help accessibility and understanding of accompanying literature. Like all infodemiology research the nature of researching online health information is limited by the constantly changing, revising and updating of online material. This study may have different results if repeated at another time.

## Conclusion

The majority of webpages relating to COVID-19 are not at a universal reading level in four major English speaking regions. However, reassuringly most webpages originated from public health organisations and government bodies. While there is an urgency in a global pandemic to publish guidance and health information, there is an onus on publishers from all information sources to publish information that is readable for all levels of comprehension, which will in turn lead to better levels of education and adherence to guidance.

## Data Availability

The datasets used and analysed during the current study are available from the corresponding author on reasonable request.

## Appendix 1

**Table 2:** List of Webpages returned after using the search terms coronavirus, COVID and COVID-19 in the Google® search engine on 17th April 2020

**Ireland Search Results**	
**Search results for ‘COVID’**	
1. https://www.who.int/emergencies/diseases/novel-coronavirus-2019/events-as-they-happen	
2. https://www.who.int/emergencies/diseases/novel-coronavirus-2019	
3. https://www.worldometers.info/coronavirus/?	
4. https://www.rte.ie/news/coronavirus/2020/0412/1130066-coronavirus-world/	
5. https://www2.hse.ie/conditions/coronavirus/coronavirus.html	
6. https://www.hse.ie/eng/services/news/newsfeatures/covid19-updates/partner-resources/covid-19-translated-resources/	
7. https://www.gov.ie/en/news/7e0924-latest-updates-on-covid-19-coronavirus/	
8. https://www.gov.ie/en/campaigns/c36c85-covid-19-coronavirus/	
9. https://en.wikipedia.org/wiki/Coronavirus_disease_2019	
10. https://www.cdc.gov/coronavirus/2019-ncov/daily-life-coping/get-your-household-ready-for-COVID-19.html	
11. https://www.fda.gov/food/food-safety-during-emergencies/food-safety-and-coronavirus-disease-2019-covid-19	
12. https://www.health.govt.nz/our-work/diseases-and-conditions/covid-19-novel-coronavirus/covid-19-current-situation/covid-19-current-cases/covid-19-significant-clusters	
13. https://www.google.com/covid19/	
14. https://www.health.harvard.edu/diseases-and-conditions/covid-19-basics	
15. https://twitter.com/hashtag/Covid19	
16. https://enterprise-ireland.com/en/About-Us/Services/Covid-19/Supports/	
17. https://www.canada.ca/en/public-health/services/diseases/coronavirus-disease-covid-19.html	
18. https://www.theguardian.com/society/2020/apr/12/domestic-violence-surges-seven-hundred-per-cent-uk-coronavirus	
19. https://www.oie.int/scientific-expertise/specific-information-and-recommendations/questions-and-answers-on-2019novel-coronavirus/	
20. https://www.healthline.com/coronavirus	
**Search results for ‘COVID-19’**	
1. https://www2.hse.ie/conditions/coronavirus/coronavirus.html	
2. https://www.gov.ie/en/campaigns/c36c85-covid-19-coronavirus/	
3. https://www.gov.ie/en/service/0039bc-view-the-covid-19-coronavirus-dashboard-showing-the-latest-stats-and/	
4. https://www.rte.ie/news/coronavirus/2020/0412/1130066-coronavirus-world/	
5. https://www.rte.ie/news/coronavirus/2020/0410/1129892-virus-strain-begins-to-show/	
6. https://www.citizensinformation.ie/en/health/covid19/	
7. https://www.hpsc.ie/a-z/respiratory/coronavirus/novelcoronavirus/	
8. https://services.mywelfare.ie/en/topics/covid-19-payments/	
9. https://en.wikipedia.org/wiki/Coronavirus_disease_2019	
10. https://enterprise-ireland.com/en/About-Us/Services/Covid-19/Supports/	
11. https://www.sfi.ie/funding/funding-calls/covid19-rapid-response/	
12. https://www.adobe.com/ie/covid-19-response.htmlhttp://www.inis.gov.ie/en/INIS/Pages/COVID-19-updates-and-announcements	
13. http://www.artscouncil.ie/Funds/Arts-Council-COVID-19-Crisis-Response-Award/	
14. https://www.ryanair.com/ie/en/useful-info/disruptions-and-refunds/coronavirus-covid-19	
15. https://microfinanceireland.ie/loan-packages/covid19/https://www.education.ie/covid19	
16. https://www.northernsound.ie/covid-19-death-toll-continues-rise/	
17. https://www.mentalhealthireland.ie/get-support/covid19/	
18. https://dbei.gov.ie/en/What-We-Do/Supports-for-SMEs/COVID-19-supports/Government-supports-to-COVID-19-impacted-businesses.html	
**Search results for ‘Coronavirus’**	
1. https://www2.hse.ie/conditions/coronavirus/coronavirus.html	
2. https://www.gov.ie/en/news/7e0924-latest-updates-on-covid-19-coronavirus/	
3. https://www.rte.ie/news/coronavirus/2020/0411/1129974-coronavirus-ireland/	
4. https://www.rte.ie/news/us/2020/0411/1129968-coronavirus-new-york-vulnerable/	
5. https://www.hpsc.ie/a-z/respiratory/coronavirus/	
6. https://www.worldometers.info/coronavirus/country/ireland/	
7. https://www.worldometers.info/coronavirus/	
8. https://www.irishtimes.com/news/health/coronavirus	
9. https://www.fsai.ie/faq/coronavirus.html	
10. https://www.theguardian.com/uk-news/live/2020/apr/12/coronavirus-live-news-nhs-staff-deaths-boris-johnson-latest-updates	
11. https://www.independent.ie/world-news/coronavirus/	
12. https://www.dfa.ie/travel/travel-advice/coronavirus/	
13. https://www.ecdc.europa.eu/en/coronavirus	
14. https://en.wikipedia.org/wiki/2019%E2%80%9320_coronavirus_pandemic	
15. https://www.who.int/emergencies/diseases/novel-coronavirus-2019	
16. https://www.ryanair.com/ie/en/useful-info/disruptions-and-refunds/coronavirus-covid-19	
17. https://www.irishmirror.ie/all-about/coronavirus-1	
18. https://www.mirror.co.uk/news/uk-news/coronavirus-live-uk-updates-covid-21839286	
19. https://www.thejournal.ie/coronavirus/news/	
20. https://www.bbc.com/news/health-51048366	
**United Kingdom Search Results**	
**Search results for ‘COVID’**	
1. https://www.worldometers.info/coronavirus/	
2. https://www.who.int/emergencies/diseases/novel-coronavirus-2019/events-as-they-happen	
3. https://www.gov.uk/government/publications/guidance-to-employers-and-businesses-about-covid-19	
4. https://www.gov.uk/coronavirus	
5. https://www.nhs.uk/conditions/coronavirus-covid-19/advice-for-people-at-high-risk/	
6. https://www.england.nhs.uk/statistics/statistical-work-areas/covid-19-daily-deaths/	
7. https://111.nhs.uk/covid-19/	
8. https://www.cdc.gov/coronavirus/2019-ncov/cases-updates/index.html	
9. https://en.wikipedia.org/wiki/Coronavirus_disease_2019	
10. https://www.standard.co.uk/news/health/coronavirus-uk-live-latest-updates-cases-toll-easter-a4412716.html	
11. https://www.google.com/covid19/	
12. https://www.bbc.com/news/world-us-canada-52249963	
13. https://www.cam.ac.uk/research/news/covid-19-genetic-network-analysis-provides-snapshot-of-pandemic-origins	
14. https://www.sciencemag.org/news/2020/04/survivors-severe-covid-19-beating-virus-just-beginning	
15. https://www.theguardian.com/membership/2020/apr/11/trump-coronavirus-crisis-dc-reporting	
16. https://www.theguardian.com/society/2020/apr/12/domestic-violence-surges-seven-hundred-per-cent-uk-coronavirus	
17. https://covid.joinzoe.com/	
18. https://twitter.com/hashtag/Covid19	
19. https://www.dailymail.co.uk/news/article-8210401/Iceland-finds-half-population-asymptomatic-infected-Covid-19.html	
20. https://covidmutualaid.org/	
**Search results for ‘COVID-19’**	
1. https://www.nhs.uk/conditions/coronavirus-covid-19/	
2. https://www.gov.uk/coronavirus	
3. https://www.gov.uk/government/publications/guidance-to-employers-and-businesses-about-covid-19	
4. https://en.wikipedia.org/wiki/Coronavirus_disease_2019	
5. https://www.who.int/news-room/q-a-detail/q-a-coronaviruses	
6. https://111.nhs.uk/covid-19/	
7. https://www.england.nhs.uk/statistics/statistical-work-areas/covid-19-daily-deaths/	
8. https://www.ecdc.europa.eu/en/covid-19-pandemic	
9. https://twitter.com/hashtag/Covid19	
10. https://www.standard.co.uk/news/health/coronavirus-uk-live-latest-updates-cases-toll-easter-a4412716.html	
11. https://www.theguardian.com/society/2020/apr/12/domestic-violence-surges-seven-hundred-per-cent-uk-coronavirus	
12. https://www.theguardian.com/membership/2020/apr/11/trump-coronavirus-crisis-dc-reporting	
13. https://news.sky.com/topic/covid-19-8518	
14. https://www.nice.org.uk/covid-19	
15. https://www.worldometers.info/coronavirus/?	
16. https://www.cam.ac.uk/research/news/covid-19-genetic-network-analysis-provides-snapshot-of-pandemic-origins	
17. https://www.cam.ac.uk/topics/covid-19	
18. https://www.independent.co.uk/topic/covid-19	
19. https://www.futurelearn.com/courses/covid-19-critical-care-education-resource	
20. https://bestpractice.bmj.com/topics/en-gb/3000168	
**Search results for ‘Coronavirus’**	
1. https://www.nhs.uk/conditions/coronavirus-covid-19/	
2. https://www.gov.uk/guidance/coronavirus-covid-19-information-for-the-public	
3. https://www.gov.uk/coronavirus	
4. https://www.worldometers.info/coronavirus/	
5. https://www.who.int/health-topics/coronavirus#tab=tab_1	
6. https://www.theguardian.com/uk-news/live/2020/apr/12/coronavirus-live-news-nhs-staff-deaths-boris-johnson-latest-updates	
7. https://www.bbc.com/news/uk-52256169	
8. https://www.bbc.com/news/world-europe-52226763	
9. https://111.nhs.uk/covid-19/	
10. https://www.mirror.co.uk/news/uk-news/coronavirus-live-uk-updates-covid-21839286	
11. https://en.wikipedia.org/wiki/Coronavirus	
12. https://en.wikipedia.org/wiki/2019%E2%80%9320_coronavirus_pandemic	
13. https://www.telegraph.co.uk/global-health/science-and-disease/coronavirus-uk-news-death-toll-boris-johnson-lockdown-extended/	
14. https://services.parliament.uk/bills/2019-21/coronavirus.html	
15. https://www.thesun.co.uk/news/11283723/coronavirus-live-updates-uk-lockdown/	
16. https://www.independent.co.uk/topic/coronavirus	
17. https://news.sky.com/topic/coronavirus-8483	
18. https://www.dailymail.co.uk/news/coronavirus/index.html	
**Canada Search Results**	
**Search results for ‘COVID’**	
1. https://www.canada.ca/en/public-health/services/diseases/coronavirus-disease-covid-19.html	
2. https://www.who.int/emergencies/diseases/novel-coronavirus-2019/events-as-they-happen	
3. https://news.ontario.ca/opo/en/2020/4/ontario-takes-further-action-to-stop-the-spread-of-covid-19.html	
4. https://www.worldometers.info/coronavirus/	
5. https://nationalpost.com/news/canada/that-is-a-surprise-doctors-still-waiting-for-feared-surge-of-covid-19-patients-in-canadian-icus	
6. https://www.cdc.gov/coronavirus/2019-ncov/daily-life-coping/get-your-household-ready-for-COVID-19.html	
7. http://www.bccdc.ca/health-info/diseases-conditions/covid-19	
8. https://www.google.com/covid19/	
9. https://www.princeedwardisland.ca/en/topic/covid-19	
10. https://www.fda.gov/news-events/press-announcements/coronavirus-covid-19-update-fda-authorizes-blood-purification-device-treat-covid-19	
11. https://www.ctvnews.ca/health/coronavirus/retired-ontario-doctor-returns-to-work-to-help-with-covid-19-1.4892573	
12. https://calgary.ctvnews.ca/1-more-death-from-covid-19-in-calgary-zone-69-new-cases-of-the-illness-in-alberta-1.4892384	
13. https://myhealth.alberta.ca/journey/covid-19/Pages/COVID-Self-Assessment.aspx	
14. https://www.sciencemag.org/news/2020/04/survivors-severe-covid-19-beating-virus-just-beginning	
15. https://bc.thrive.health/	
16. https://globalnews.ca/news/6806734/coronavirus-canada-prison-cases/	
17. https://www.npr.org/sections/goatsandsoda/2020/04/09/830297538/scientists-try-to-figure-out-if-summer-will-slow-the-spread-of-covid-19	
18. https://www.health.govt.nz/our-work/diseases-and-conditions/covid-19-novel-coronavirus/covid-19-current-situation/covid-19-current-cases/covid-19-significant-clusters	
19. https://twitter.com/hashtag/covid%E3%83%BC19?lang=en	
20. https://nymag.com/intelligencer/2020/04/life-after-covid-the-view-from-beijing.html	
**Search results for ‘COVID-19’**	
1. https://www.canada.ca/en/public-health/services/diseases/coronavirus-disease-covid-19.html	
2. https://www.canada.ca/en/public-health/services/diseases/2019-novel-coronavirus-infection.html	
3. https://www.alberta.ca/coronavirus-info-for-albertans.aspx	
4. https://news.ontario.ca/opo/en/2020/4/ontario-takes-further-action-to-stop-the-spread-of-covid-19.html	
5. http://www.bccdc.ca/health-info/diseases-conditions/covid-19	
6. https://nationalpost.com/news/canada/that-is-a-surprise-doctors-still-waiting-for-feared-surge-of-covid-19-patients-in-canadian-icus	
7. https://www.gov.mb.ca/covid19/index.html	
8. https://www2.gov.bc.ca/gov/content/safety/emergency-preparedness-response-recovery/covid-19-provincial-support	
9. https://www.albertahealthservices.ca/topics/Page16944.aspx	
10. https://www.princeedwardisland.ca/en/topic/covid-19	
11. https://www.ctvnews.ca/health/coronavirus/phone-data-reveals-who-is-staying-home-during-covid-19-1.4892194	
12. https://www.camh.ca/en/health-info/mental-health-and-covid-19	
13. https://www.gov.nl.ca/covid-19/	
14. https://globalnews.ca/tag/covid-19/	
15. https://en.wikipedia.org/wiki/Coronavirus_disease_2019	
16. https://www.quebec.ca/en/health/health-issues/a-z/2019-coronavirus/situation-coronavirus-in-quebec/	
17. https://www.intact.ca/en/covid19.html	
18. https://www.toronto.ca/home/covid-19/	
19. https://sharedhealthmb.ca/covid19/screening-tool/	
20. https://www.halton.ca/For-Residents/Immunizations-Preventable-Disease/Diseases-Infections/New-Coronavirus	
**Search results for ‘Coronavirus’**	
1. https://www.canada.ca/en/public-health/services/diseases/2019-novel-coronavirus-infection/symptoms.html	
2. https://www.canada.ca/en/public-health/services/diseases/coronavirus-disease-covid-19.html	
3. https://nationalpost.com/news/world/two-thirds-of-acute-coronavirus-cases-tested-on-antiviral-drug-improved	
4. https://globalnews.ca/news/6808593/coronavirus-bc-ferries-easter-henry/	
5. https://globalnews.ca/news/6809091/snl-coronavirus-tom-hanks/	
6. https://www.worldometers.info/coronavirus/	
7. https://www.thestar.com/coronavirus.html	
8. https://www.bbc.com/news/health-51048366	
9. https://www2.gnb.ca/content/gnb/en/departments/ocmoh/cdc/content/respiratory_diseases/coronavirus.html	
10. https://novascotia.ca/coronavirus/	
11. https://www.alberta.ca/coronavirus-info-for-albertans.aspx	
12. https://www.aljazeera.com/topics/events/coronavirus-outbreak.html	
13. https://www.aljazeera.com/news/2020/01/countries-confirmed-cases-coronavirus-200125070959786.html	
14. https://www.ontario.ca/page/how-your-organization-can-help-fight-coronavirus	
15. https://www.gov.mb.ca/covid19/index.html	
16. https://www.quebec.ca/en/health/health-issues/a-z/2019-coronavirus/	
17. http://www.bccdc.ca/about/news-stories/stories/2020/information-on-novel-coronavirus	
18. https://www.who.int/emergencies/diseases/novel-coronavirus-2019	
19. https://www.albertahealthservices.ca/topics/Page16944.aspx	
20. https://www.bdc.ca/en/pages/special-support.aspx	
**United States of America Search Results**	
**Search results for ‘COVID’**	
1. https://www.cdc.gov/coronavirus/2019-ncov/daily-life-coping/get-your-household-ready-for-COVID-19.html	
2. https://www.cdc.gov/coronavirus/2019-ncov/covid-data/covidview/index.html	
3. https://www.who.int/emergencies/diseases/novel-coronavirus-2019/events-as-they-happen	
4. https://www.worldometers.info/coronavirus/	
5. https://www.dhs.wisconsin.gov/covid-19/index.htm	
6. https://floridahealthcovid19.gov/	
7. https://www.doh.wa.gov/Emergencies/Coronavirus	
8. https://www.ncdhhs.gov/divisions/public-health/covid19	
9. https://en.wikipedia.org/wiki/Coronavirus_disease_2019	
10. https://www.osha.gov/Publications/OSHA3990.pdf	
11. https://www.cbsnews.com/live-updates/coronavirus-pandemic-covid-19-latest-news-2020-04-10/	
12. https://www.osha.gov/SLTC/covid-19/	
13. https://www.mass.gov/resource/information-on-the-outbreak-of-coronavirus-disease-2019-covid-19	
14. https://www.mass.gov/info-details/covid-19-updates-and-information	
15. https://coronavirus.jhu.edu/	
16. https://www.google.com/covid19/	
17. https://coronavirus.ohio.gov/wps/portal/gov/covid-19/home	
18. https://covid19.nj.gov/	
19. https://www.apple.com/covid19/	
20. https://covid19.ca.gov/	
**Search results for ‘COVID-19’**	
1. https://www.cdc.gov/coronavirus/2019-ncov/index.html	
2. https://www.cdc.gov/coronavirus/2019-ncov/cases-updates/cases-in-us.htm	
3. https://www.who.int/emergencies/diseases/novel-coronavirus-2019/events-as-they-happen	
4. https://www.who.int/emergencies/diseases/novel-coronavirus-2019	
5. https://www.dhs.wisconsin.gov/covid-19/index.htm	
6. https://www.osha.gov/SLTC/covid-19/	
7. https://floridahealthcovid19.gov/	
8. https://en.wikipedia.org/wiki/Coronavirus_disease_2019	
9. https://www.scdhec.gov/infectious-diseases/viruses/coronavirus-disease-2019-covid-19	
10. https://www.google.com/covid19/	
11. https://www.ncdhhs.gov/divisions/public-health/covid19	
12. https://www.worldometers.info/coronavirus/?utm_campaign=homeAdvegas1?	
13. https://www.mass.gov/resource/information-on-the-outbreak-of-coronavirus-disease-2019-covid-19	
14. https://www.mass.gov/info-details/covid-19-updates-and-information	
15. https://www.cdph.ca.gov/Programs/CID/DCDC/Pages/Immunization/ncov2019.aspx	
16. https://covid19.nj.gov/	
17. https://www.apple.com/covid19/	
18. https://coronavirus.ohio.gov/wps/portal/gov/covid-19/home	
19. https://www.cbsnews.com/live-updates/coronavirus-pandemic-covid-19-latest-news-2020-04-10/	
20. https://eu.usatoday.com/story/news/health/2020/04/11/coronavirus-us-more-covid-19-deaths-than-italy-any-country/5121962002/	
**Search results for ‘Coronavirus’**	
1. https://www.cdc.gov/coronavirus/2019-ncov/index.html	
2. https://www.cdc.gov/coronavirus/2019-ncov/symptoms-testing/symptoms.html	
3. https://www.who.int/health-topics/coronavirus#tab=tab_1	
4. https://www.worldometers.info/coronavirus/	
5. https://www.coronavirus.gov/	
6. https://www.nytimes.com/2020/04/12/world/coronavirus-news.html	
7. https://www.cbsnews.com/live-updates/coronavirus-pandemic-covid-19-latest-news-2020-04-10/	
8. https://www.cbsnews.com/live-updates/coronavirus-pandemic-covid-19-latest-news-2020-04-11/	
9. https://www.michigan.gov/coronavirus/	
10. https://en.wikipedia.org/wiki/Coronavirus	
11. https://portal.ct.gov/coronavirus	
12. https://edition.cnn.com/2020/04/11/health/us-coronavirus-updates-saturday/index.html	
13. https://www.foxnews.com/category/health/infectious-disease/coronavirus	
14. https://www.huffpost.com/news/topic/coronavirus?guccounter=1&guce_referrer=aHR0cHM6Ly93d3cuZ29vZ2xlLmNvbS8&guce_referrer_sig=AQAAAJAxqRSywUja50843Nl6I0EH84GQoeWD0l_2u-c0eXdmpvmP6-giGX7SeLPjo6udRrN6QpN-lpBYJxuy5tplY6cJ300Tp-LQmmIGNPezvtJFWoSVFwaY8oY9thd5JiqxA7b541a5N7JwzzTCh2y5C3oVwK9-6THdDritxInILu8R	
15. https://www.aljazeera.com/news/2020/01/coronavirus-symptoms-vaccines-risks-200122194509687.html	
16. https://www.nbcnews.com/health/coronavirus	
17. https://www.washingtonpost.com/gdpr-consent/?next_url=https%3a%2f%2fwww.washingtonpost.com%2fhealth%2f2020%2f02%2f28%2fwhat-you-need-know-about-coronavirus%2f	
18. https://www.dshs.state.tx.us/coronavirus/	
19. http://www.vdh.virginia.gov/coronavirus/	
20. https://www.usa.gov/coronavirus	

## Appendix 2

**Table 3:** Spearman Correlation results for Inter-Readability Tool Analysis. Results given are formatted as: r value, *p* value, 95% confidence interval

	FRES	FKG Median (range)	GFI Median (range)	SMOG Median (range)
**FRES**		−0.774, *p* < 0.0001, 95% CI [− 0.8216 to − 0.7161]	−0.267, *p* < 0.0001, 95% CI [− 0.3848 to − 0.1426]	−0.355, *p* < 0.0001, 95% CI [− 0.4646 to − 0.2364]
**FKG**	−0.774, *p* < 0.0001, 95% CI [− 0.8216 to − 0.7161]		0.728, *p* < 0.0001, 95% CI [0.6613 to 0.7846]	0.791, *p* < 0.0001, 95% CI [0.7373 to 0.8357]
**GFI**	−0.267, *p* < 0.0001, 95% CI [− 0.3848 to − 0.1426]	0.728, *p* < 0.0001, 95% CI [0.6613 to 0.7846]		0.909, *p* < 0.0001, 95% CI [0.8842 to 0.9298]
**SMOG**	−0.355, *p* < 0.0001, 95% CI [− 0.4646 to − 0.2364]	0.791, *p* < 0.0001, 95% CI [0.7373 to 0.8357]	0.909, *p* < 0.0001, 95% CI [0.8842 to 0.9298]	

## Abbreviations

ANOVA: Analysis of variance
COVID-19: Coronavirus Disease 2019
FKG: Flesch Kinaid Grade
FRES: Flesch Reading Ease Score
GFI: Gunning Fox Index
SD: Standard Deviation
SMOG: Simple Measure of Gobbledygook
